# The unified state qualification exam STEP-1 as a marker of the success of the education of pediatric doctors in Ukraine and Bogomolets National Medical University

**DOI:** 10.1186/s12909-024-05261-0

**Published:** 2024-03-08

**Authors:** Nataliya V. Obernikhina, Lesya V. Yanitska, Oksana V. Vygovska

**Affiliations:** 1 Department of Medical Biochemistry and Molecular Biology, Bogomolets National Medical University, 13 T. Shevchenko boul., 01601 Kyiv, Ukraine; 2 Dean of the Faculty “Pediatrics” Bogomolets, National Medical University, 13 T. Shevchenko boul., 01601 Kyiv, Ukraine

**Keywords:** Higher medical education, Specialty "Pediatrics", USQE STEP-1, Bogomolets NMU, Medical biochemistry

## Abstract

**Background:**

Ukraine’s higher medical education goes deeper and deeper every year in the European integration processes in the field of «Health Care» knowledge. Since 2005, the integrated license integrated exam STEP “General medical training” has been introduced in the country to diagnose the quality of training of specialists in all medical specialties. Since 2019, Ukraine, unlike other countries in Europe and the world, has been training specialists in the specialty “Pediatrics” at the stage of undergraduate training. The quality control of the training of specialists is carried out in the form of passing the Unified State Qualification Exam STEP (USQE STEP) separately for each medical specialty (Medicine and Pediatrics). Therefore, the purpose of our research is to conduct a comparative analysis of the results of the success of the first stage of the USQE STEP-1 by students of higher medical education in the specialty “Pediatrics” with the specialty “Medicine” in Ukraine and in the Bogomolets National Medical University (Bogomolets NMU).

**Methods:**

Analytical references to the results of the first stage of the USQE STEP-1 for the students who have completed theoretical medical disciplines specialty “Pediatrics” and the specialty “Medicine” in Ukraine and Bogomolets NMU, which are provided by the Testing Center at the Ministry of Health of Ukraine. Тhe statistical significance of comparative indicators was proved using Fisher’s test, with a statistical error that corresponded to the specified value for ≤ 0.05.

**Results:**

It is shown that in 2022, applicants of higher medical education of Ukraine with the specialty “Pediatrics” improved the overall success rate by 8.4%, and the success rate of subtests by an average of 10.5%, despite the state of war in Ukraine. The exception was the results of the licensing exam for the subtest component “Biochemistry”: compared to 2021, the pass rate decreased by 3.6% in the specialty “Medicine” and by 6.4% in the specialty “Pediatrics”. At Bogomolets NMU, the leaders of 2022 were the students of the “Pediatrics” specialty, their success rate is 2% higher than that of the “Medicine” specialty.

**Conclusions:**

The analysis of the results of USQE STEP-1 by applicants of higher medical education of the specialties “Pediatrics” and “Medicine” in Ukraine showed the effectiveness of the selection of the specialty “Pediatrics” into a separate section of the training of specialists at the undergraduate level in the field of “Health Care”. Using the methods of mathematical statistics, the effectiveness of organizational methodological techniques in the organization of the educational process in the conditions of the martial law of Ukraine and Bogomolets NMU as a leader in the training of specialists in Pediatric doctors has been proved.

**Supplementary Information:**

The online version contains supplementary material available at 10.1186/s12909-024-05261-0.

## Introduction

The quality of training of specialists of high professional level in the field of medicine directly depends on the objective assessment of their skills to use the acquired knowledge in the process of obtaining the university degree. The world practice of using the state standardized system of external assessment of the professional competence of professionals in the field of healthcare has been proving its effectiveness for many decades. Such systems of testing are introduced in the USA (The United States Medical Licensing Exam (USMLE) [1]. All graduates of medical schools in India pass the Foreign Medical Graduate Examination (FMGE) conducted by the National Board of Examination (NBE); the procedure and the result of the examination are supervised by the Medical Council of India (MCI), which is now known as the National Medical Commission (NMC) [2]. In the UK, the General Medical Council (GMC) conducts state licensing exams for future doctors, consisting of professional and linguistic parts, testing for specialists who graduated in other countries (Professional and Linguistic Assessments Board - PLAB) is also introduced, thus confirming the level of qualification of doctors [3]. Graduates of medical institutions of the EU countries pass such exams as CMC MCQ and clinical exam CMC (country medical council) [4, 5]. Any of these systems is based on Multiple Choice Question with one correct answer [6, 7].

The methodological basis of the technology of test examinations in Ukraine is the state standards of higher education, the requirements for which are determined by the Resolution of the Cabinet of Ministers of Ukraine dated from August 7, 1998 № 1247 “About the development of state standards of higher education,” which are consistent with the international standards ISO and EC and Standards for Educational and Psychological Testing [8]. A system similar to USMLE was introduced in Ukraine in 2003 - the Krok (eng. - Step) licensing exam for the specialty “Medicine,” since 2005 there has been a distribution of licensing exams for the specialties “Pharmacy” and “Dentistry” [8, 9]. The World and European Medical School trains specialists in medical, dental specialties, special attention is paid to applicants in the specialty “Pharmacy”. “Pediatrics” is only a section of the specialty “Medicine,” despite the fact that specialists study separately a number of disciplines with the characteristics of children’s development, including obstetrics and gynecology [10, 11].

Since 2019, the Ministry of Health of Ukraine has been amended to conduct the KROK licensing exam in the form of the Unified State Qualification Exam (USQE) for applicants for higher education of the second level “Master” of the field of knowledge “Healthcare,” separating specialty “Pediatrics.” The components and stages of the Unified State Examination are defined in the Resolution of the Cabinet of Ministers dated from March 28, 2018 № 334 “About the Approval of the Procedure for the Implementation of the Unified State Examination for Applicants of the Second-Level Higher Education Degree “Master” in the specialties of the field of knowledge “Health Care” [12]. So, according to the adopted changes, the first stage takes place in the third year of study of applicants for higher education and involves passing the integrated test exam STEP-1 and the exam in English of a professional direction. The purpose of the first stage is to evaluate the ability to use knowledge and understanding of key concepts of fundamental biomedical sciences, such as biology (6–8% of test tasks of the total), normal anatomy (9–11%), histology (4–6%), normal physiology (13–17%), biological chemistry (13–17%), pathological physiology (13–17%) pathological anatomy (10–14%), microbiology (7–9%) [8]. It is important to note that in the USMLE licensing exam system the content of test assignments in Biological Chemistry is up to 24% of the total, in the MCI system - up to 16%, in Europe and the UK - up to 14%. This percentage makes it possible to pay attention to the principles and mechanisms underlying the preservation of health, the identification of the disease and models of its treatment. Mastering of these fundamental disciplines by applicants involves the formation in future specialists the understanding of the basics of the functioning of the human body in normal conditions and under pathological changes, which is extremely important for the implementation of knowledge and skills during professional activity, the competencies of which are checked at the stage of passing the second stage of the USQE. The second stage consists of an integrated STEP-2 exam and a clinical exam, the purpose of which is to assess the level of professional competence of applicants in professionally oriented (clinical) disciplines. Thus, applicants for higher medical education of all specialties will be assessed according to uniform requirements and they must correspond to a high level of competence.

It should be noted that in European countries the specialization “Pediatrics” is prestigious, although it is not separated in the system of medical education as an individual specialty. The peculiarity of training specialists in the specialty “Pediatrics” abroad is postgraduate education, that is, after graduating from the Faculty of General Medicine, the residency in pediatrics continues for five years, during which children’s cardiology, cardiac surgery, neurosurgery, allergology, surgery, etc. are the most popular in European countries [13, 14]. In Ukraine the training of pediatricians takes place at the pre-diploma stage, and the internship in pediatrics lasts for 2 years. The educational and professional training program for specialists in the specialty “Pediatrics” consists of basic and optional components, unlike the specialty “Medicine”, each component includes topics and questions devoted to the peculiarities of childhood and development (see *Supplementary materials*, S2-3). Therefore, the special professional competencies of a pediatrician are formed during the entire training cycle and can be tested at the intermediate level of quality control of higher medical education as USQE STEP-1 specialty “Pediatrics”. This is a significant difference in the form of training of specialists in specialty “Pediatrics,” in which Ukraine has an advantage.

### The purpose of the study

to conduct a comparative analysis of the results of the first stage of the USQE STEP-1 by students of higher medical educational institutions specializing in “Pediatrics” in Ukraine during 2019–2022 as a national indicator of the success of the selection of the specialty “Pediatrics” into a separate section of training specialists at the undergraduate level in the field of health care health. To compare the obtained success results with the same ones for the “Medicine” specialty to understand the quality of the work of higher medical education institutions in Ukraine. Separately to conduct a comparative analysis of the results of the first stage of the USQE STEP-1 by students at the Bogomolets National Medical University a Medical School that trains most of the pediatric doctors of Ukraine.

## Materials and methods

Basic disciplines in Ukraine are studied by students of higher medical educational institutions in the specialty “Medicine” and specialty “Pediatrics” during the first three years of study. Their list and load in ECTS credits are the same for both specialties. Regarding the specifics of the content of the disciplines of the “Pediatrics” specialty, it is indicated above. Therefore, an intermediate evaluation of the level of training of specialists can be done based on the results of USQE STEP-1. The first stage of the exam is taken by students after the successful completion of the 6th semester of study, that is, both men and women of the same age category. At the beginning of the exam, 4,509 applicants for the specialty “Medicine” were registered, of which 843 were applicants from Bogomolets NMU, and 222 applicants for the specialty “Pediatrics”, of which 126 were applicants from Bogomolets NMU. More detailed information about the demographic composition of test participants can be obtained from the State Non-Profit Enterprise Testing Board for Professional Competence Assessment of Higher Education Trainees in Medicine and Pharmacy at the Ministry of Public Health of Ukraine (Testing Center) [8].

The research materials are the analytical references to the results of exams provided by the State Non-Profit Enterprise Testing Board for Professional Competence Assessment of Higher Education Trainees in Medicine and Pharmacy at the Ministry of Public Health of Ukraine [8] for the specialties “Medicine” and “Pediatrics” in Ukraine and Bogomolets NMU.

In the course of the analysis, the following were calculated: arithmetic mean indicators of the results of the first stage of the USQE STEP-1 in Ukraine and Bogomolets NMU by both national indicator and subtests; correlation coefficient between average arithmetic indicators of the results of the first stage of the USQE STEP-1 Bogomolets NMU and the all-Ukrainian indicator for specialties “Medicine” and “Pediatrics”; correlation coefficient between the indicators of success of the first stage of the USQE STEP-1 according to the subtest “Biological Chemistry” of Bogomolets NMU and specialties “Medicine” and “Pediatrics.”

Statistical significance of comparative indicators is proved using Fisher’s F-test [15–17] with a statistical error that corresponded to a given value for ≤ 0,05. This made it possible to determine the level of reliability of the compared indicators in the period from 2019 to 2022 and their dependence on the factors of the educational process.

## Results

### Analysis of the results of the USQE STEP**-**1 in Ukraine by applicants for higher medical education (HME) specialty “Pediatrics” in 2022

In Ukraine there are only 6 institutions of higher education (IHEs) that train specialists in the specialty “Pediatrics”: Vinnytsia National Medical University (VNMU), Ivano-Frankivsk National Medical University (IFNMU), Lviv National Medical University (LNMU), Bogomolets National Medical University (Bogomolets NMU), Kharkiv National Medical University (KNMU), Sumy State University (SSU). For the first stage of the USQE in 2022, according to the data of the institutions of higher education, 236 applicants were registered, 222 applicants have passed the USQE, 150 of them are the applicants for budgetary (67.6%) and 72 are the applicants (32.4%) for contractual forms of education. The criterion of success “passed” was set at the level of 62.0% [8]. The contingent of applicants and the results of the first stage of the USQE STEP-1 are given in Table 1.

**Table 1:** The results of the success of the first stage of the USQE STEP-1 in Ukraine in specialty “Pediatrics”

№	IHEs	Amount of applicants that passed STEP-1			% of total	Amount of applicants that didn’t pass STEP-1	
		Total	Budgetary form	Contractual form		Total	%
1	NMU	126	79	47	56,8	21	16,7
2	VNMU	34	26	8	15,3	1	2,9
3	IFNMU	23	18	5	10,4	4	17,4
4	LNMU	22	20	2	9,9	3	13,6
5	SSU	10	0	10	4,5	2	20,0
6	KNMU	7	7	0	3,2	0	0,0
	**Total**:	**222**	**150**	**72**	**100,0**	**31**	**14,0**

**Fig. 1:**
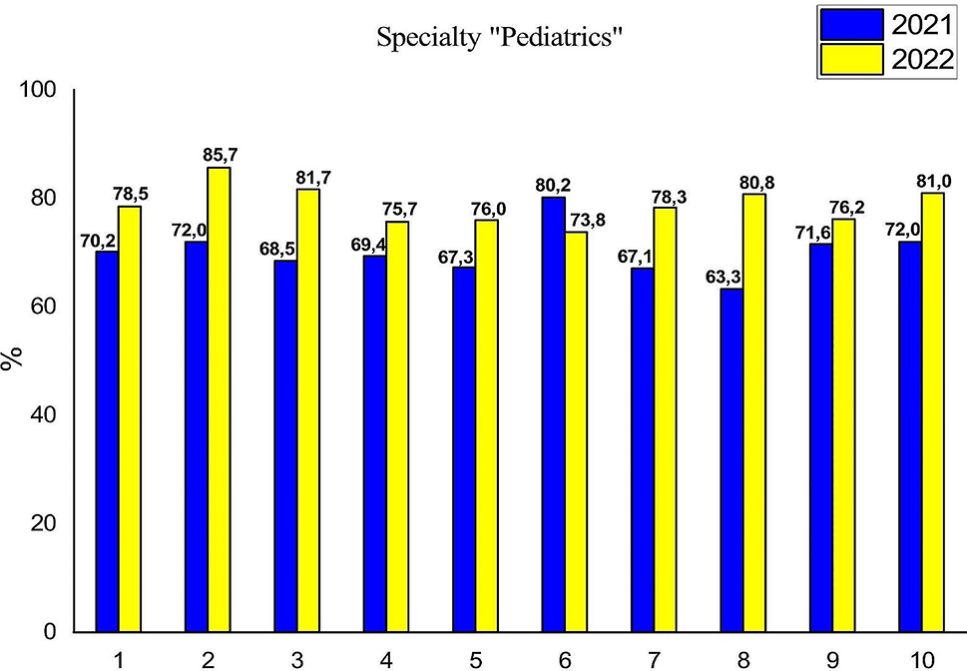
Results of the first stage of the USQE STEP-1 by applicants HME in specialty “Pediatrics” in Ukraine: 1– national average, 2– biology, 3– normal anatomy, 4– histology, 5– normal physiology, 6– biochemistry, 7– pathological physiology, 8– pathological anatomy, 9– microbiology, 10– pharmacology

We will analyze the dynamics of educational success indicators for specialty “Pediatrics” according to subtests formed in percentage correspondence of fundamental biomedical sciences, demonstrated in Fig. 1.

Let’s consider the success rates of applicants for higher medical education Bogomolets NMU in specialty “Pediatrics” and conduct a comparative analysis of the results with specialty “Medicine.”

### Analysis of the results of the USQE STEP-1 by applicants HME bogomolets NMU

We will analyze the results of the first stage of the USQE STEP-1 by applicants of higher medical education of Bogomolets NMU for the specialty “Pediatrics” in the context of passing the licensing exam in Ukraine during 2019–2022, the results of the analysis are given in Table 2.

**Table 2:** Comparison of the results of the success of the first stage of the USQE STEP-1, 2019–2022

Specialties, years Success indicators	2019	2020	2021		2022	
	*common*	*common*	*«Medicine»*	*«Pediatrics»*	*«Medicine»*	*«Pediatrics»*
NI, %	71,2	74,1	72,1	70,2	80,1	78,5
^a^GI_NMU_, %	76,0	75,3	69,9		76,3	
^b^GI_NMU-SP_, %	68,8	65,3	71,2	67,4	76,7	78,4

^a^GI_NMU_ is a general success indicator at Bogomolets NMU

^b^GI_NMU-SP_ is a general success indicator at Bogomolets NMU in the specialties of “Pediatrics” and “Medicine”

### Analysis of the results of the USQE STEP-1 by applicants HME bogomolets NMU specialty “Pediatrics” by subtests

**Fig. 2:**
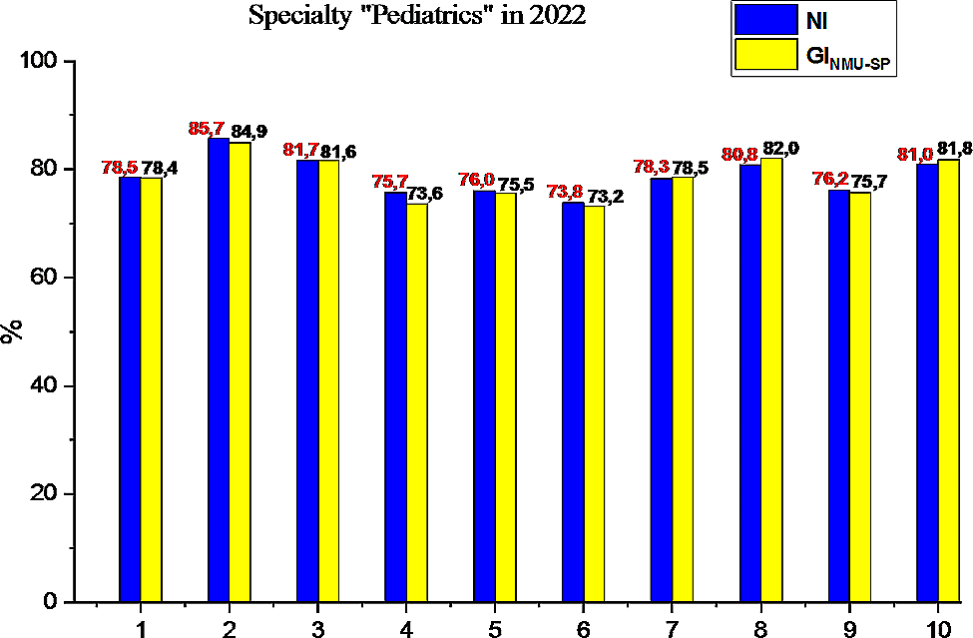
Results of the first stage of the USQE STEP-1 by applicants HME in specialty “Pediatrics” in Ukraine and Bogomolets NMU: 1– national average, 2– biology, 3– normal anatomy, 4– histology, 5– normal physiology, 6– biochemistry, 7– pathological physiology, 8– pathological anatomy, 9– microbiology, 10– pharmacology

In the process of ensuring the possibility of creating flexible and individual ways of obtaining knowledge in a higher medical school against the background of reducing the learning time of each component of fundamental biomedical sciences [18–20], we will analyze the results of the USQE STEP-1 by subtests, graphically presented in Fig. 2.

**Fig. 3:**
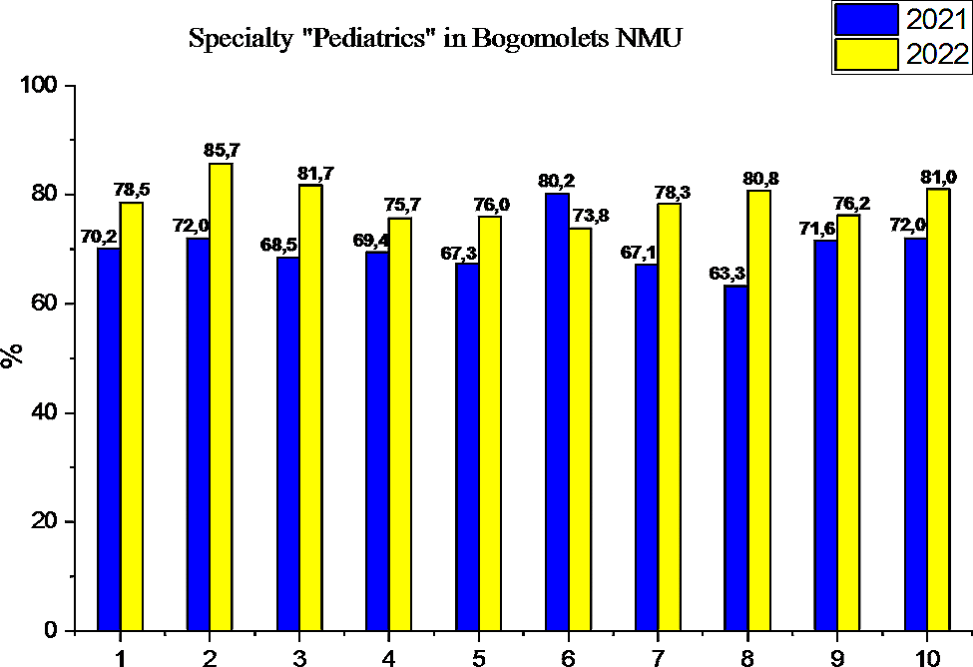
Comparison of the results of the USQE STEP-1 by applicants HME in specialty “Pediatrics” Bogomolets NMU: 1– Bogomolets NMU average, 2– biology, 3– normal anatomy, 4– histology, 5– normal physiology, 6– biochemistry, 7– pathological physiology, 8– pathological anatomy, 9– microbiology, 10– pharmacology

Let’s consider the success of the first stage of the USQE STEP-1 by applicants for HME of Bogomolets NMU in the specialty “Pediatrics” by subtests in comparison with 2021, presented in Fig. 3.

In order to prove the assumption of the impact of improving the methodological methods of passing a stable educational process according to the mixed form of training for applicants for higher medical education in specialty “Pediatrics” (2019) on the results of the success of indicators in the conditions of martial law (2022), methods of mathematical statistics were used, namely the Fisher F-criterion and the calculation of variances [15, 16]. Additionally, an analysis of the comparison of the parameters of the general populations was carried out, formula 1 [16] was used to calculate the variance value. The results of calculations of variances of indicators of 2019 and 2022 of specialty “Pediatrics” are given in Table 3.

**Table 3:** Results of variance calculations for the NI, GI_NMU_ and GI_NMU−SP “Pediatrics”_ indicators

	Indicators	Variance calculations
**1**	NI	0.52400697
**2**	GI_NMU_	0.52172345
**3**	GI_NMU−SP “Pediatrics”_	0.55886065

The null hypothesis assumes that these samples are independent and are taken from general populations with the same variances with a significance coefficient of α = 0.05. Then, σ 2 will theoretically have a value of 0.5, with the deviation region being 1.96. Analysis of the calculations shows that the values of the indicators are within the limits of variance, so we can say that the NI, the general success indicator of Bogomolets NMU and the success indicator of “Pediatrics” Bogomolets NMU in 2022 are likely and logical. We will calculate the Fisher’s criterion, the results of which are presented in Table 4.

**Table 4:** Results of F-criterion calculations for the NI, GI_NMU_ and GI_NMU−SP “Pediatrics”_ indicators

	Indicators	F-criterion indicator to compare with F_krit_ (1.6–1.0)	
		F_emp_-2019	F_emp_-2022
**1**	NI	1.09	1.18
**2**	GI_NMU_	1.01	1.02
**3**	GI_NMU−SP “Pediatrics”_	1.06	1.12

**Fig. 4:**
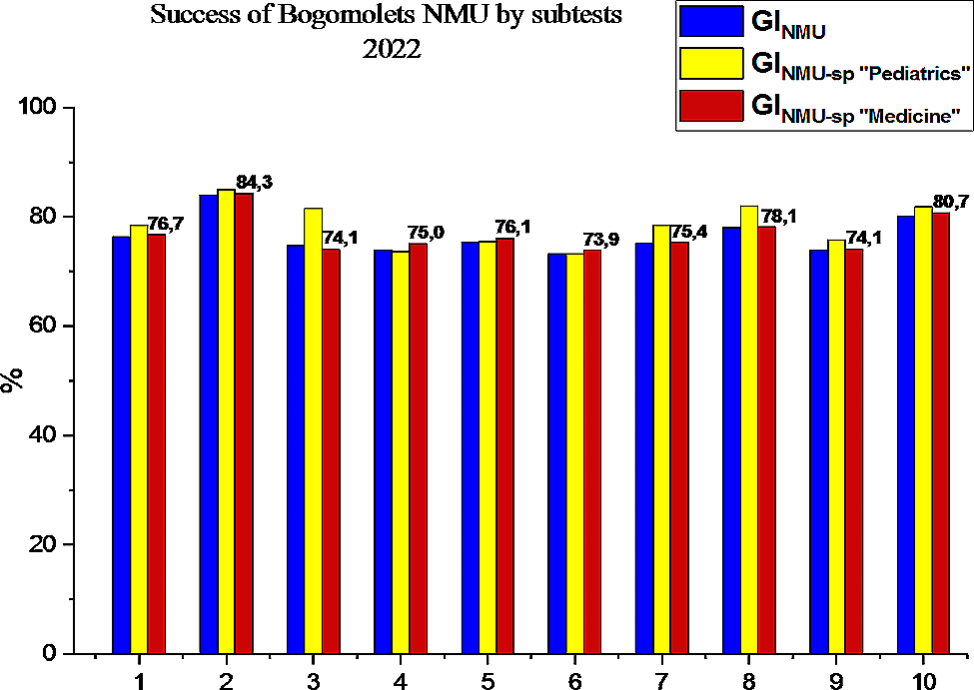
Comparison of the results of the USQE STEP-1 by applicants HME in the specialties “Pediatrics” and 222 “Medicine” in Bogomolets NMU by subtests: 1– Bogomolets NMU average, 2– biology, 3– normal anatomy, 4– histology, 5– normal physiology, 6– biochemistry, 7– pathological physiology, 8– pathological anatomy, 9– microbiology, 10– pharmacology

So, consider the success of the first stage of the USQE STEP-1 by applicants for HME of Bogomolets NMU in the specialty “Pediatrics” by subtests in comparison with specialty “Medicine” in 2022, the results are shown in Fig. 4.

### Analysis of the results of subtests on Biological Chemistry as a component of the USQE STEP-1 by applicants HME in specialty “Pediatrics”

**Fig. 5:**
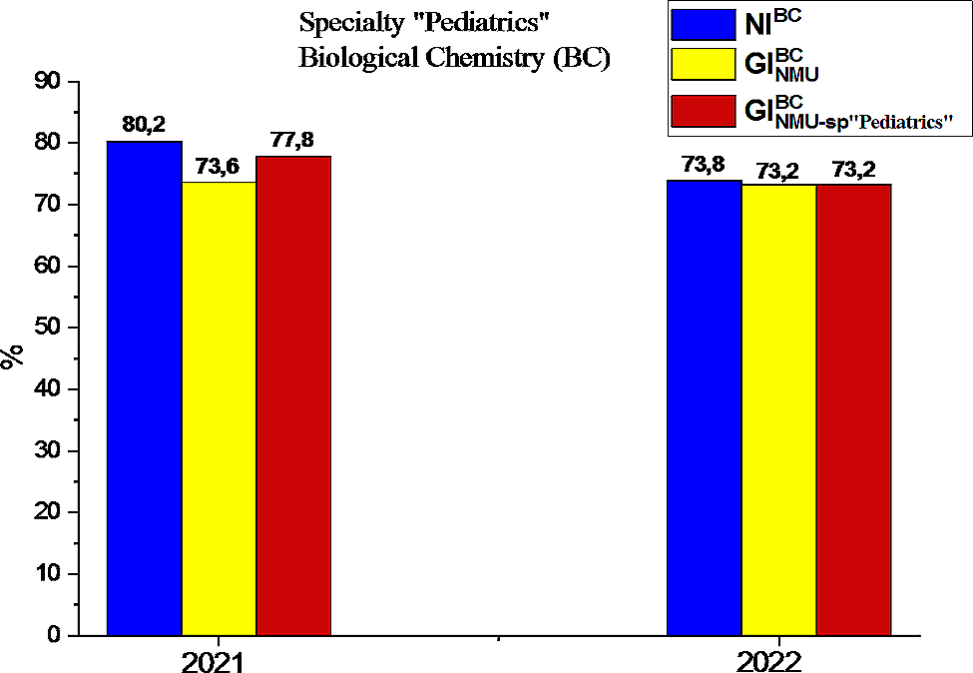
Comparison of the results of the USQE STEP-1 in biological chemistry by applicants HME the specialty “Pediatrics”

The results of the USQE STEP-1 by applicants HME in Ukraine and Bogomolets NMU specialty “Pediatrics” by subtest subtest “Biological chemistry” are among the worst in 2022, so we will conduct an analysis of their success, presented on the Fig. 5.

In order to prove the assumptions regarding the results of passing biological chemistry, methods for comparing indicators using the Fisher’s criterion were used [15], a table of F-values was analyzed by methods of comparing the numerical data of the criterion [16, 17]. According to the null hypothesis, the difference in the success of test tasks passing by applicants HME specialty “Pediatrics” of the Bogomolets NMU does not depend on teaching methods, but is the result of the influence of martial law in the country on applicants. An alternative hypothesis was the assumption of the level of complexity of the test tasks of the USQE STEP-1 by the subtest component in biological chemistry. The results of calculating the variances of indicators in biological chemistry of the specialty “Pediatrics” in 2021 and 2022 are given in Table 5.

**Table 5:** Results of variance calculations for the NI^BC^, GI^BC^_NMU_ and GI^BC^_NMU−SP “Pediatrics”_ indicators

	Indicators	Variance calculation
**1**	NI^BC^	0.46052143
**2**	GI^BC^_NMU_	0.40403742
**3**	GI^BC^_NMU−SP “Pediatrics”_	0.40403742

The null hypothesis assumes that the determined samples are independent and taken from general populations with the same variances with a significance coefficient of α = 0.05. Then, σ 2 theoretical will have a value of 0.48, while the deviation area is 1.96. According to the results of the analysis of Table 5, we can assert that the values of the variance calculations of the indicators of the success of passing of the USQE STEP**-**1 in Biological Chemistry are not included in the boundaries of the variance, therefore we can say that the success results in terms of NI^BC^ numbers, the general indicator of Bogomolets NMU (GI^BC^_NMU_) and the general indicator of Bogomolets NMU “Pediatrics” (GI^BC^_NMU-SP “Pediatrics”_) in 2022 were random and not logical. The results of calculating the F-criterion of the indicators that have been already mentioned are given in Table 6.

**Table 6:** Results of calculating the F-criterion for the NI^BC^, GI^BC^_NMU_ and GI^BC^_NMU−SP “Pediatrics”_ indicators

	Indicators	F-criterion indicator to compare with F_krit_ (1.6–1.0)	
		F_emp_-2021	F_emp_-2022
**1**	NI^BC^	1.07	1.16
**2**	GI^BC^_NMU_	1.02	1.26
**3**	GI^BC^_NMU−SP “Pediatrics”_	1.02	1.26

## Discussion

The global epidemic of COVID-19 forced the educational space to react quickly. Therefore, in a very short time, the education system switched to a remote form of providing educational services. The quality of this form of education is extremely important for training future doctors, especially during the separation specialty “Pediatrics” period. Therefore, as shown in Table 1, the improvement of methodological methods of passing a stable educational process in the mixed form of education of students of higher medical education in the specialty “Pediatrics” turned out to be quite effective. Bogomolets National Medical University provides the country with specialists in specialty “Pediatrics” for 56.8% of the total number of applicants, and all other IHEs train 43.2% of specialists. As Table 1 shows, only 31 applicants out of 222 applicants for higher medical education in specialty “Pediatrics” did not pass the first stage of the USQE STEP-1 from the first compilation, which is 14% of the total number of applicants achieved success. The highest results of the first stage of the USQE STEP-1 in terms of the overall success rate were demonstrated by KNMU and VNMU, in contrast to SSU with a success rate of 20%. The national success indicator (NI) of the first stage of the USQE STEP-1 for the specialty “Pediatrics” in 2022 is 78.5%, for the specialty “Medicine” NI is 80.1% [8], so it means that the level of training of specialists in the specialty “Pediatrics” within Ukraine is quite high.

However, since 2022, Ukraine has been under martial law. The vast majority of participants in the educational process were not ready for such events. Some applicants temporarily emigrated abroad, some remained in the occupied territories, some - in the territories of active and moderate hostilities. During this period, the physical factors of war had an impact: air alarms, shelters without access to the Internet, lack of light and mobile communications. A tangible impact on the participants of the educational process was on the part of psychological factors of war, such as fear, despair, confusion, etc. In such circumstances, of course, the educational process suffers the greatest losses in terms of acquiring knowledge. Thus, the educational environment continues to use and improve forms of education, adapting to the conditions of full-scale war. Therefore, the results of such work will be reflected in the indicators of passing of the USQE STEP-1. Despite the martial law in Ukraine, the success of applicants for higher medical education in the specialty “Pediatrics” in 2022 increased both by NI (∼ by 8%) and the national indicator by subtests (NIS): biology by 13.7%, normal anatomy by 13.2%, histology by 6.3%, normal physiology by 8.7%, pathological physiology by 11.2%, pathological anatomy by 17.5%, microbiology by 4.6%, pharmacology by 9%. As we can see in Fig. 1, the exception is the result of the passing of biological chemistry, which has deteriorated in the country by 6.4%. Therefore, on average, the success of applicants by subtest improved by 8.6% compared to 2021.

We can note that during the transition from offline education (indicators of 2019) to mixed form of education due to the COVID-19 pandemic (2020–2021 p.p.), the scientific and pedagogical team of Bogomolets NMU took all the necessary measures to ensure the mixed form of the educational process, and such changes did not significantly affect the quality of knowledge assimilation by students (see Table 2 and [9, 18]). The NI of the first STEP-1 licensing exam by applicants for higher medical education in Ukraine improved by 3.1% in 2020, the GI_NMU_ rates of Bogomolets NMU slightly worsened despite the fact that the criterion of success “passed” in 2020 was set at the lowest level in the history of licensing exams: 55.5%, and the country was in a transitional state of organizing the educational process of mixed studying due to the COVID-19 pandemic. It should be noted that the GI_NMU−SP_ of applicants for the specialty “Pediatrics” at Bogomolets NMU in 2020 is lower by 11.9% compared to specialty “Medicine” [9]. In 2021, the criterion of success “passed” in Ukraine was set at 60.0%, the results of the first stage of the USQE STEP-1 in Ukraine and in Bogomolets NMU worsened by 3% and 5.4%, respectively, but the success of applicants for specialty “Pediatrics” improved by 2.1% compared to the previous year, reaching the period of offline education [18].

With the beginning of martial law, Ukraine in the educational process was forced to implement a mixed, mixed form of education, industry “Health” was not an exception. However, the success (GI_NMU-SP_) of the first stage of the USQE STEP-1 by applicants of higher medical education of specialties “Pediatrics” and “Medicine” in Ukraine improved by 8% and 8.3%, respectively, and the general success in Bogomolets NMU (GI_NMU_) improved by 6.4%, despite the establishment of the criterion of success “passed” at the level of 62.0%. Although the national indicator of the country of specialty “Pediatrics” is 1.6% lower than the NI of specialty “Medicine,” in Bogomolets NMU applicants of specialty “Pediatrics” passed the license exam STEP-1 by 1.7% more successfully. We can say that Ukrainian applicants are very motivated, conscious citizens of their country.

According to the standardized table the F-criterion (Fkrit) [16, 17] of the study results has a value from 1.6 to 1.0. Analysis of the results presented in Table 4 indicates that the F-criterion values prove the reliability of the results obtained, since the Femp values for 2019 (1.01–1.09) and Femp for 2022 (1.02–1.18) are within the probability range according to the theoretical value table Fkrit. Thus, the results obtained in 2019 and 2022 are within the limits of reliability. Therefore, the provision of the educational process in the mixed form of education in Ukraine and the Bogomolets NMU was carried out at the proper methodological level. Even during air raids, classes in medical and theoretical disciplines continue in bomb shelters (see *Supplementary material*, S4). In the Bogomolets NMU students who did not have time to arrive remain in the shelters and connect to the group on special online educational platforms developed by institutions of higher medical education, where they perform all the tasks together with their colleagues and, if necessary, consult with the teacher through video communication. A lot of attention among tasks is given to test control.

Fundamental biomedical sciences form the basis for higher medical education applicants for clinical reasoning and decision-making to solve new, complex and ambiguous clinical problems that require a deeper knowledge fund. A detailed understanding of the biomedical sciences also allows future doctors to effectively exploit the innovations and discoveries resulting from basic scientific research [19–23]. The success of applicants for higher medical education in the specialty “Pediatrics” of Bogomolets NMU, as Fig. 2 shows, in 2022 is almost at the level of the state indicator of Ukraine, and for such subtests as pathological anatomy and pharmacology, the Bogomolets NMU GI_NMU-SP_ slightly (∼ by 1.2%) exceeds NI. Having conducted a detailed analysis of the results of the first stage of the USQE STEP-1 by applicants for higher medical education in the specialty “Pediatrics” Bogomolets NMU by subtests, we note that the in 2022 success rate increased both in the general indicator of Bogomolets NMU by 11%, (3% higher than the NI of the country), and by subtests: biology by 16.6% (3% higher than the country’s NI), normal anatomy by 17.9% (4.7% higher than the country’s NI), histology by 7.0%, normal physiology by 11.6% (3% higher than the country’s NI), pathological physiology by 13.7% (2.5% higher than the country’s NI), pathological anatomy by 20.8% (3.3% higher than the country’s NI), microbiology by 7.0% (2.4% higher than the country’s NI), pharmacology by 11.1% (2.1% higher than the country’s NI). Despite the significant success of passing the first stage of the USQE STEP-1 in almost all subtests, the result of biological chemistry in applicants for higher medical education in specialty “Pediatrics” of Bogomolets NMU in 2022 worsened by 4.6%, which is 1.8% less than in Ukraine (see Fig. 1). The results of the first stage of the USQE STEP-1 by applicants for higher medical education in specialty “Medicine” at Bogomolets NMU are approximately at the same level of success as the general success indicator of Bogomolets NMU, and from such subtests as histology, normal physiology and biochemistry are slightly higher (see Fig. 4). Of such subtests as biology, normal anatomy, pathological physiology, pathological anatomy, microbiology, pharmacology, the candidates for specialty “Pediatrics” remain the leader. Therefore, the Bogomolets National Medical University demonstrated a high level of preparation of applicants the specialty “Pediatrics” for the USQE STEP-1 at a level higher than the state indicator.

Biological chemistry as one of the main disciplines forms the understanding of the structure, properties, functions, the use of biological compounds from micro- and macromolecules to cells to understand their role in life, the occurrence of various kinds of disorders, as well as the understanding of the mechanisms of influence of drugs, their function in clinical conditions [24–26]. In the process of ensuring the possibility of creating flexible and individual ways of obtaining knowledge in a higher medical school against the background of reducing the learning time of each component of the fundamental biomedical sciences [19, 25, 26], we will analyze the results of the first stage of the USQE STEP-1 by subtests, paying special attention to biological chemistry, the preparation for which is carried out by the department of medical biochemistry and molecular biology at Bogomolets NMU.

The passing rate of Biological Chemistry by applicants HME the specialty “Pediatrics” in 2022 according to the national indicator (NI) decreated by 6.4% (see Fig. 5). However, the results of the Bogomolets NMU (GI_NMU_) in all medical specialties remained at the level of 2021 [18]. After analyzing the results of the USQE STEP-1 in Biological Chemistry by applicants HME the specialty “Pediatrics” in Bogomolets NMU (GI_NMU−SP “Pediatrics”_), we see that the success rate has decreased by 4.6% compared to 2021, but this indicator is not lower than the GI_NMU_. That is, a decrease NI^BC^ in biological chemistry against the background of a general increase in the results of passing the first stage of the USQE STEP-1 specialty “Pediatrics” (see Fig. 1) is most likely associated with an increase in the level of complexity of test tasks in the licensed exam compared to 2021 and with the factor of hostilities in Ukraine in conditions of constant deep stress, which primarily affects memory and attention. Since memory is able to perceive bright events at once, however, after thirty minutes our memory turns off and the perception of any information is blocked [26]. Analysis of the indicators given in Table 6 shows that the F-criterion numbers prove the reliability of the obtained results, since the F_*emp*_ numbers for 2021 (1.02–1.07) and F_*emp*_ numbers for 2022 (1.16–1.26) are within probability, according to the theoretical numbers of the F-criterion(F_*krit*_) [17, 18]. Thus, the results obtained in 2021 and 2022 belong to the limits of probability, but the results of the success of biological chemistry indicators for 2021 are more reliable. Thus, we can state that the effectiveness of training future doctors of specialty “Pediatrics” depends on the use of improved methodological techniques in the organization of the educational process in the discipline “Medical Biochemistry,” introduced by the Department of Medical Biochemistry and Molecular Biology of the Bogomolets National Medical University, namely, the video convention of the discipline was developed, the forms of control of theoretical knowledge and practical skills were diversified, the test tasks for the topics of the course “Medical Biochemistry” were updated and improved. Тhe results of success in 2022 were random, associated with the impact of external conditions, namely the state of war in which Ukrainian medical education is located. The reliability of the conclusions is proved by methods of mathematical statistics, in particular by reliability indicators.

## Conclusions

The analysis of the results of USQE STEP-1 by applicants of higher medical education of the specialties “Pediatrics” and “Medicine” in Ukraine showed the effectiveness of the selection of the specialty “Pediatrics” into a separate section of the training of specialists at the undergraduate level in the field of “Health Care”. Using the methods of mathematical statistics, the effectiveness of organizational methodological techniques in the organization of the educational process in the conditions of the martial law of Ukraine and Bogomolets NMU as a leader in the training of specialists in Pediatric doctors has been proved.

### Electronic supplementary material

Below is the link to the electronic supplementary material.

Supplementary Material 1

## Data Availability

The datasets used and/or analyzed during the current study available from the corresponding author on reasonable request. Supplementary material is NOT available on the publisher’s website along with the published article.

## References

[1] Chaudhry HJ, Katsufrakis PJ, Tallia AF (2020). The USMLE Step 1 decision: an opportunity for Medical Education and Training. JAMA.

[2] Tandon N, Misra KS, Mallikarjuna C, Chhina RS, Chandra H, Sudha S, Puri B, Basu SN, Randeep Guleria R (2023). With active contribution from the distinguished members of the governing body of NBEMS. Natl Board Exam J Med Sci.

[3] Smajdor A, Herring J, Wheeler R. ‘Doctors and the General Medical Council (GMC)’, *Oxford Handbook of Medical Ethics and Law*. 2021; 201–210. 10.1093/med/9780199659425.003.0019.

[4] Kovacs E, Schmidt AE, Szocska G, Busse R, McKee M, Legido-Quigley H (2014). Licensing procedures and registration of medical doctors in the European Union. Clin Med (Lond).

[5] De Lange S (2001). The European Union of medical specialists and speciality training. Eur J Anaesthesiol.

[6] Park J (2010). Constructive multiple-choice testing system. BJET.

[7] Lakshmanan M (2019). Effect of negatively framed MCQS in the medical education. World J Pharm Res.

[8] Tsentr testuvannya pry Ministerstvi. okhorony zdorovʺya Ukrayiny. Kyyiv: DO «Tsentr testuvannya». URL: https://www.testcentr.org.ua/uk.

[9] Obernikhina N, Sanzhur T, Kramarenko I, Hayova L (2020). Krok-1. Medicine in Bogomolets National Medical University as education indicator. High Educ Res.

[10] Hedgepeth D, Wlasowicz S, Lott R, Smith T (2023). The early impact of deciding to take the United States Medical Licensing Examination Step 1 for Osteopathic Medical students in the Pass/Fail era. Cureus.

[11] Roberts WL, Gross GA, Gimpel JR, Smith LL, Arnhart KP, Xiaomei YA. June 16,. (2020). An Investigation of the Relationship Between COMLEX-USA Licensure Examination Performance and State Licensing Board Disciplinary Actions. *Acad. Med* 2020; 95(6): 925–930. 10.1097/ACM.0000000000003046. 10.1097/ACM.0000000000003046 31626002

[12] https://zakon.rada.gov.ua/laws/show/334-2018-%D0%BF#Text.

[13] Rideout M, Dawlett M, Plant J, Chitkara M, Trainor JL (2021). Essential yet Ill-defined: leadership roles to support fourth-year medical students in pediatrics. Med Educ Online.

[14] Hejda G, Mazur A, Dembiński Ł, Peregud-Pogorzelski J, Jackowska T, Walczak M, Szczepański T (2020). Healthcare for children and adolescents in Poland. Turk Pediatri Ars.

[15] Graham JGU (1992). Fisher’s exact test. J R Stat Soc Ser Stat Soc.

[16] https://dspace.uzhnu.edu.ua/jspui/handle/lib/34910.

[17] erpub.chnpu. edu.ua:8080/jspui/handle/123456789/7058.

[18] Dickinson BL, Gibson K, VanDerKolk K, Greene J, Rosu CA, Navedo DD, Porter-Stransky KA, Graves LE (2020). It is this very knowledge that makes us doctors: an applied thematic analysis of how medical students perceive the relevance of biomedical science knowledge to clinical medicine. BMC Med Educ.

[19] Obernikhina NV, Yanitska LV, Posternak NO (2023). Analysis of the results of the Unified State qualification exam step 1 specialty «Pediatrics» as a modernization of the educational process of higher medical institutions of Ukraine. Mod Pediatr Ukraine.

[20] Cangiarella J, Cohen E, Rivera R, Gillespie C, Abramson S (2020). Evolution of an accelerated 3-year pathway to the MD degree: the experience of New York University Grossman School of Medicine. Acad Med.

[21] Schwartz CC, Ajjarapu AS, Stamy CD, Schwinn DA (2018). Comprehensive history of 3-year and accelerated US medical school programs: a century in review. Med Educ Online.

[22] Pock AR, Durning SJ, Gilliland WR, Pangaro LN (2019). Post-carnegie II curricular reform: a north American survey of emerging trends & challenges. BMC Med Educ.

[23] Popova TM, Bachinsky RO, Polishchuk TV. Innovative methods in teaching of biological chemistry. Med Clin Chem. 2020;2100–4. 10.11603/mcch.2410-681X.2020.v.i2.11367.

[24] Ishchenko A, Stuchynska N, Tolmachova V (2022). Efficiency of the methods for forming the Chemical Safety competence of future doctors. RREM.

[25] Nicolaou KC (2014). The Chemistry-Biology-Medicine Continuum and the Drug Discovery and Development process in Academia. Chem Biology.

[26] Savelyuk N (2022). Perception of the War-related stress: experience of Ukrainian students. Psychology:Reality Perspect.

