# Supplementary material for: The unified state qualification exam STEP-1 as a marker of the success of the education of pediatric doctors in Ukraine and Bogomolets National Medical University
Source: BMC Med Educ. 2024 Mar 8;24:263. doi: 10.1186/s12909-024-05261-0 (PMC10924366; doi:10.1186/s12909-024-05261-0)

Supplementary material

THE UNIFIED STATE QUALIFICATION EXAM STEP-1 AS A MARKER OF THE SUCCESS OF THE EDUCATION OF PEDIATRIC DOCTORS IN UKRAINE AND BOGOMOLETS NATIONAL MEDICAL UNIVERSITY

Nataliya V. Obernikhina[1], Lesya V. Yanitska[1], Oksana V. Vygovska[2]

[1]**Department of** Medical Biochemistry and Molecular Biology Bogomolets National Medical University, 13 T. Shevchenko boul., 01601 Kyiv, Ukraine
[2]Dean of the Faculty "Pediatrics" Bogomolets National Medical University, 13 T. Shevchenko boul., 01601 Kyiv, Ukraine

Correspondence
Nataliya V. Obernikhina, Ph.D., Associate Professor, Bogomolets National Medical University
E-mail: nataliya.obernikhina@gmail.com

Disciplines of the educational and professional program of the specialty "Pediatrics" S2-S3
https://nmuofficial.com/akredytatsiya-2022/

Training with enhanced security measures S4

Components (disciplines) of the educational and professional program of the specialty "Pediatrics" (examples)

https://nmuofficial.com/akredytatsiya-2022/

**The main components of the educational program (study subjects)**
Social and humanitarian training
English language (by professional direction)
Ukrainian language (by professional direction)
Philosophy, ethics
History of Ukraine and Ukrainian culture
Fundamental training
Medical terminology with the basics of the Latin language
Human anatomy, including features of childhood
Histology, cytology and embryology, including features of childhood
Medical biology
Medical and biological physics
Medical informatics
Medical biochemistry
Physiology, including features of childhood
Microbiology, virology (with the basics of immunology)
Molecular biology
Professional training
Occupational Health in
Hygiene and ecology
Social medicine, public health
Pathomorphology, including features of childhood
Pathophysiology, including features of childhood
Pharmacology
Propaedeutics of internal medicine, including nursing practice, basic medical skills in the therapeutic department

Elective components (disciplines)
1 course (15 credits ECTS)
Information and scientific search. Basics of the scientific research
Medical information systems
Life safety, basics of bioethics and biosafety
General military training
Physical education and health
Introduction to the specialty of pediatrics
Deontology in pediatrics. Communication skills of a pediatrician
Psychology of communicating with a child at reception
Anti-corruption and integrity
2 course (15 credits ECTS)
Scientific research and medical ethics in pediatrics
General tactics
Physical education and health
Biogenic elements and their role in a child's life
Medical embryology with the basics of reproductive and teratology
Clinical anatomy and operative surgery, including with features of childhood
Clinical physiology of the child
Nutritionology
Basic resuscitation measures in pediatrics
Psychotrauma in children and adolescents in the context of the family
Anti-corruption and integrity

Training with enhanced security measures

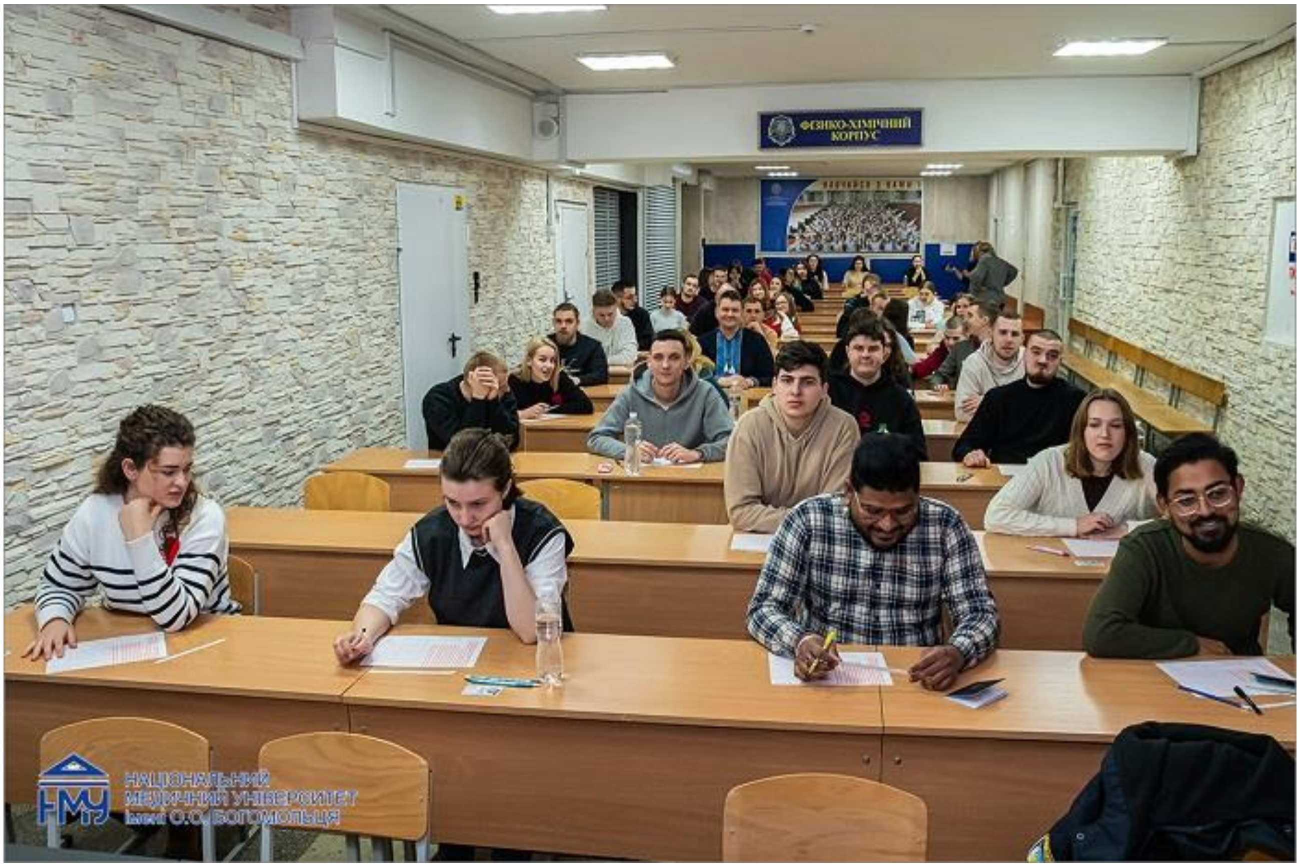

Supplement: Supplementary file 1 — Supplementary Material 1 [file 12909_2024_5261_MOESM1_ESM.pdf]
